# Predicting The Developmental Potential Of Cleavage Stage Embryos Based On Oxygen Consumption Rate In FET Cycles

**DOI:** 10.5935/1518-0557.20190080

**Published:** 2020

**Authors:** Shubiao Han, Jiying Hou, Xiaodong Zhang, Guoning Huang

**Affiliations:** 1 Chongqing Key Laboratory of Human Embryo Engineering, Chongqing Reproductive and Genetics Institute, Chongqing Health Center for Women and Children. Chongqing, China; 2 Department of Histology and Embryology, Laboratory of Stem Cells and Tissue Engineering, Chongqing Medical University. Chongqing, China

**Keywords:** oxygen consumption, FET, scanning electrochemical microscopy (SCEM), implantation rate

## Abstract

**Objective:**

To study the value of oxygen consumption (OC) as a predictor of the developmental potential of D3 embryos in frozen embryo transfer (FET) cycles.

**Methods:**

This observational study included 148 patients undergoing FET cycles with two embryos transferred per cycle. OC rates were examined by scanning electrochemical microscopy before embryo transfer. Implantation, clinical pregnancy, miscarriage, and live birth rates were calculated.

**Results:**

A total of 296 embryos were transferred in 148 cycles, or two embryos per cycle. The embryos were divided into three groups based on OC: Group A included the cases in which the OC rate of each of the two transferred embryos was greater than 3.0 fmol/s; Group B included the cases in which the OC rate of one of the embryos was greater than 3.0 fmol/s and the OC rate of the other embryo was less than 3.0 fmol/s; and Group C included the cases in which the OC rates of the two embryos were less than 3.0 fmol/s. Higher live birth rates and lower miscarriage rates were observed in Group A (*p*<0.05).

**Conclusions:**

Our data suggest that OC is positively correlated with embryo developmental potential. Therefore, measuring the OC of human embryos may be useful in embryo assessment.

## INTRODUCTION

Accurate assessment of developmentally competent embryos is crucial for the success of ART treatment. For decades, embryo quality assessment based on morphological evaluation was used to predict implantation and pregnancy rates to some degree (Gardner *et al*., 2004). Numerous alternatives to morphological assessment have been proposed to assess the quality of embryos, including methods based on spent embryo culture medium, cell cycle timing, morphological evaluation by time-lapse systems (TLSs), and PGS (Twisk *et al*., 2006; Armstrong *et al*., 2015). The above technologies have provided important insight into early embryo development and the arbitrary nature of embryo selection; however, none has become standard practice, and an early prediction of clinically relevant, quality embryos before transfer still remains challenging (Kurosawa *et al*., 2016).

Embryo evaluation based on oxygen metabolism or consumption rate has been attempted via objective and quantitative methods (Rodgaard *et al*., 2015). Oxygen consumption rate has been recently considered a metabolic indicator that reflects the activity of mitochondrial oxidative phosphorylation (OXPHOS), by which adenosine triphosphate (ATP) is synthesized. Animal experiments have described a correlation between embryo OC and developmental potential. Using a non-invasive method, Shiku *et al*. (2001) succeeded at determining oxygen consumption by individual oocytes and embryos using a measurement system based on scanning electrochemical microscopy (SECM) that enables the evaluation of mitochondrial activity in oocytes and embryos. Tejera *et al*. (2016) showed that OC is a marker for human oocyte competence. To our knowledge, few observational studies have looked into OC as a marker to predict human embryo developmental potential.

This study aimed to measure embryo OC patterns before frozen-thawed embryo transfer (FET) cycles and to explore the correlation between OC rate and embryo developmental potential.

## MATERIALS AND METHODS

### Patient and embryo selection

The study included embryos warmed between January 2015 and May 2015 at the Chongqing Reproductive and Genetics Institute. All survived and two embryos were transferred per patient in the first FET cycle. The patients were aged ≤ 37 years and had been diagnosed with tubal factor infertility. They had undergone insemination by IVF and had ≥3 D3 embryos available. No fresh embryo transfer at oocyte retrieval (OR) was included. Patients with positive preimplantation genetic diagnosis and transfers with donated gametes were excluded. All procedures were performed according to the Declaration of Helsinki on medical research involving human subjects. The Ethics Committee of the Chongqing Obstetrics and Gynecology Hospital approved the study (certificate no. RGI-ZA201408). The embryos included in the study were divided into three groups based on OC rate: Group A included the cases in which the OC rate of each of the two transferred embryos was greater than 3.0 fmol/s; Group B included the cases in which the OC rate of one of the embryos was greater than 3.0 fmol/s and the OC rate of the other embryo was less than 3.0 fmol/s; and Group C included the cases in which the OC rates of the two embryos were less than 3.0 fmol/s.

### Fresh cycle, embryo cryopreservation by vitrification

In fresh cycles, ovarian stimulation and luteal supplementation were performed according to our standard practice (Ye *et al*., 2009). A standard insemination protocol was used for all patients. In sum, 39-40 hours after hCG administration, 10,000-15,000 spermatozoa per microdrop were added to G-IVF medium pre-equilibrated overnight. The oocyte was then added to the microdrop, and co-cultured with sperm at 37℃ in 5% O_2_ and 6% CO_2_ in an incubator. The day of oocyte retrieval was defined as Day 0. Before 9 am on Day 1, the oocytes were observed after the removal of granular cells, and fertilization was confirmed by the presence of two pronuclei (PN) and two polar bodies. Embryo quality was evaluated using a morphological scoring system, which accounts for the regularity of blastomeres, degree of fragmentation, and embryo microscopic appearance, as previously described (Xiong *et al*., 2011). The embryos were cryopreserved on Day 3. The Cryotop method for embryo vitrification was as described by Kuwayama (2007). After vitrification, the Cryotop devices were plunged directly into liquid nitrogen. The embryos were further stored in vapor phase nitrogen containers.

### FET cycles

Warmed embryos were transferred in natural cycles, stimulated cycles (gonadotropin or clomiphene citrate), or hormone replacement cycles as described previously (Yamanaka *et al*., 2011). The embryos were transferred on Day 4. In order to warm the embryos, the Cryotop was removed from LN2 storage and immediately plunged into thawing solution (TS; 1.0 M sucrose in TCM199+20% SSS) at 37℃. After 1 min, the embryos were transferred to a dilution solution (DS; 0.5 M sucrose in TCM199+20% SSS) at room temperature. After 3 min, they were transferred to a washing solution (WS; TCM199+20% SSS) for 5 min twice. Subsequently, the embryos were transferred to G2.5 (Vitrolife, Sweden) medium containing 5% human serum albumin cultured overnight before transfer. Embryos with more than 50% intact blastomeres were recorded as survivors. The tools and solutions used in embryo freezing and thawing were procured from Kitazato (Kitazato BioPharma Co., Japan).

### Evaluation of embryo oxygen consumption rates and embryo transfer

After overnight culture (20-24h), the oxygen consumption rate of individual embryos was quantified on Day 4 before transfer with a modified SECM measuring system described in the literature (Yamanaka *et al*., 2011). In sum, the two embryos of each patient were transferred into a well filled with modified TCM199+20% SSS medium. The embryos sank to the bottom of the cone-shaped microwell and remained at its lowest point, each microwell containing one embryo. A platinum microdisk electrode was loaded into 5 ml of modified TCM199+20% SSS medium, and its tip potential was maintained at -0.6 V *versus* Ag/AgCl with a potentiostat to monitor local oxygen concentration. The microelectrode scanned along the z-axis from the edge of each embryo, and the oxygen consumption rate was calculated with custom software based on spherical diffusion theory. Oxygen levels were measured in about two minutes. After oxygen consumption rates were measured, the two embryos were returned to culture medium and then transferred to the patients. Clinical pregnancy was established when a gestational sac was detected by transvaginal ultrasound 28 days after embryo transfer.

### Statistical analysis

The data sets were analyzed on software package Stata (Stata 10.0 version, STATA Inc., College Station, TX). Patient data were expressed as mean ± SD for purposes of comparison. Quantitative data were compared using the t-test. Categorical variables were analyzed via the chi-square test. A *p* value<0.05 was considered to be statistically significant.

## RESULTS

A total of 296 embryos from 148 transfer cycles met the inclusion criteria. One hundred and seventy embryos from 85 cycles were included in Group A; 72 embryos from 36 cycles were included in Group B; and 54 embryos from 27 cycles were included in Group C. Table 1 describes patient demographic and baseline characteristics. The demographic characteristics of the patients submitted to frozen-thawed embryos transfers were not significantly different in terms of age, body mass index (BMI), number of oocytes retrieved, number of available embryos, mean blastomere number, or endometrial thickness.

**Table 1:** Demographic and baseline characteristics of the patients in the three groups

IU	A	B	C	*p* value^a^	*p* value^b^
No. cycles	85	36	27	-	-
age	30.04±3.48	29.47±3.68	30.33±3.60	0.419	0.709
BMI	21.74±2.33	20.83±2.51	21.28±3.07	0.057	0.411
No. oocytes retrieved	9.90±2.89	10.00±2.79	10.09±2.89	0.860	0.764
Mean blastomere number	10.32±2.77	10.08±2.72	10.29±2.75	0.662	0.961
No. available embryos	3.56±1.88	3.74±1.90	3.63±1.83	0.632	0.866
Mean endometrial thickness (mm)	10.05±0.90	10.17±1.16	10.14±1.06	0.541	0.666

a Group A *vs*. Group B

b Group A *vs*. Group C

Table 2 shows implantation, clinical pregnancy, miscarriage, and live birth rates. The three groups were not significantly different in terms of implantation, pregnancy, or miscarriage rates (50.00% *vs.* 45.83% *vs.* 40.74, *p*>0.05; 67.06% *vs.* 50.00% *vs.* 51.85%, *p*>0.05; 4.07% *vs.* 11.11% *vs.* 14.81%, *p*>0.05). However, Group A yielded significantly higher live birth rates than Groups B and C (56.47% *vs.* 36.11% *vs.* 33.33%, *p*<0.05).

**Table 2:** Clinical outcomes from the three groups

	A	B	C	*p* value^a^	*p* value^b^
No. cycles	85	36	27	-	-
Implantation rate (%)	50.0 % (85/170)	45.83% (33/72)	40.74% (22/54)	0.576	0.275
Pregnancy rate (%)	67.06% (57/85)	50.00%(18/36)	51.85%(14/27)	0.101	0.174
Miscarriage rate (%)	4.07%(4/85)	11.11% (4/36)	14.81% (4/27)	0.236	0.094
Live birth rate (%)	56.47%(48/85)	36.11%(13/36)	33.33% (9/27)	0.048	0.047

a Group A vs. Group B

b Group A vs. Group C

## DISCUSSION

Oxygen consumption rate has been recently considered a metabolic indicator that reflects the activity of mitochondrial oxidative phosphorylation (OXPHOS), by which adenosine triphosphate (ATP) is synthesized (Kurosawa *et al*., 2016). Therefore, mitochondrial dysfunctions may lead to inferior embryo development and lower pregnancy rates from IVF cycles. According to the literature, respiration increases indicate mitochondrial development from the morula to the blastocyst stage in most animal embryos, while embryos with high oxygen consumption rates provide for good viability and freezing ability. In addition, it has been established that embryos with above average respiration rates also yield higher pregnancy rates. These results demonstrate that oxygen consumption is a good parameter to assess embryo quality.

OC rate detected by SECM has often been regarded as a better indicator of overall metabolic activity (Tejera *et al*., 2011). It has also been demonstrated that oxygen consumption is related to oocyte fertilization capability, which is consistent with previous data regarding higher ATP turnover in oocytes (Ruvolo *et al*., 2013). Yamanaka *et al*. (2011) found that the OC rate might be used to select vitrified-warmed blastocysts with high developmental potential. Yoshida *et al.* (2013) found that when the oxygen consumption rate increased in a group of thawing embryos, their rate of blastocyst development and freezing ability for cryopreservation increased. Tejera *et al*. (2012) found that OC rates during embryo development were positively associated with implantation potential and embryo quality; therefore, OC measurements during embryo culture - performed almost immediately after embryo transfer - were deemed to add value to the process of selecting viable embryos.

This observational study was designed to test how effectively OC may be used as a predictor of embryo developmental potential, and to investigate the relationship between OC and pregnancy outcomes. Our study was based solely on FET cycles. The OC rates of each of the 296 embryos included in the study were calculated before embryo transfer. The embryos were divided into three groups based on OC: Group A included the cases in which the OC rate of each of the two transferred embryos was greater than 3.0 fmol/s; Group B included the cases in which the OC rate of one of the embryos was greater than 3.0 fmol/s and the OC rate of the other embryo was less than 3.0 fmol/s; and Group C included the cases in which the OC rates of the two embryos were less than 3.0 fmol/s. The three groups were compared for implantation, clinical pregnancy, miscarriage, and live birth rates. Transfers of two embryos with OC rates greater than 3.0 fmol/s yielded higher live birth rates, a finding that situates the OC rate as a reliable predictor of human cleavage-stage embryo developmental potential.

OC showed a positive correlation with embryo developmental potential. Embryo OC measurement based on SECM enabled the assessment of respiration of single human embryos. Our findings and current literature on the subject indicates that this might become an alternate method for embryo selection in IVF laboratories. Randomized controlled trials are required to further validate the existing evidence.

### Authors’ roles

Han Shubiao and Huang Guoning designed the study. Hou Jiying participated in the experiment. Zhang Xiaodong analyzed the data. Han Shubiao and Zhang Xiaodong wrote the paper.
